# Knowledge domain, research hotspots and frontiers in physiology teaching reforms from 2012 to 2021: A bibliometric and knowledge-map analysis

**DOI:** 10.3389/fmed.2023.1031713

**Published:** 2023-03-20

**Authors:** Jia Xu, Shimeng Sun, Yadong Zhao, Qing Ma

**Affiliations:** ^1^Department of Physiology and Pharmacology, NBU Health Science Center, Ningbo University, Ningbo, Zhejiang, China; ^2^The First Affiliated Hospital of Ningbo University, Ningbo, Zhejiang, China

**Keywords:** bibliometrics, knowledge-map, physiology, teaching reform, visualization

## Abstract

**Objective:**

To identify author collaborations and impact; participating countries, institutions, and journals; evaluate the knowledge base; and analyze research hotspots and frontiers in teaching reforms in physiology.

**Methods:**

Articles and reviews related to teaching reforms in physiology published between January 1, 2012, and December 31, 2021, were obtained from the Web of Science Core Collection. Two Scientometric software applications (CiteSpace 5.7 and VOSviewer 1.6.15) were used to perform bibliometric and knowledge-map analysis, generate network maps, and identify research trends and top keywords, authors, co-cited authors, institutions, countries, journals, and references.

**Results:**

The search identified a total of 2,882 papers in 466 academic journals by 13,895 authors from 4,072 organizations in 67 countries/regions. Physiology teaching reform-related publications increased rapidly over time. Arango-Lasprilla and Rivera published the most papers, while Moseley had the most co-citations. Active collaborations among physiology researchers were noted. *Advances in Physiology Education* published the most papers on physiology teaching reforms and was also the top co-cited journal in the Medicine/Medical/Clinical, Psychology/Education/Health, and Neurology/Sports/Ophthalmology fields. The United States and University of California published the most physiology teaching publications in the search criteria. Ten references (research articles and reviews) on mechanisms and diseases were identified as the knowledge base. The mainstream research directions were education, Alzheimer’s disease, performance, physiology, and risk factors. Mental health and emotion regulation are increasing in significance and may become new hotspots. The research trend to move from the field of pain pathogenesis to the field of neuropsychiatry has become increasingly clear. This tendency away from peripheral system-based disorders to central system-based orders is inextricably linked to further developments in physiological understanding of the brain.

**Conclusion:**

This study analyzed the research hot spots and frontiers of teaching reforms on in physiology using bibliometric and visual methods. Based on the results, rehabilitation, neurosciences, and infectious disease are hot topics in physiology. In particular, the pathogenesis of neurological diseases, treatment strategies, and technology updates have gradually become research hotspots. We predict that this trend is closely related to the implementation of brain research programs in various countries. These findings provide helpful references for scholars focusing on physiology education.

## Introduction

Physiology is the study of the body and its functional activities (1). Physiology also explores the intrinsic relationship between such activities and the occurrence, development, and treatment of diseases, and is thus a cornerstone for clinical medicine and research as well as an important bridge between basic and clinical disciplines (2). The Nobel Prize in physiology and medicine (3–5) is considered the most prestigious in the world and reflects the importance of this topic. Accordingly, physiology is a compulsory subject for undergraduate medical majors and for the National Professional Licensing Examination and the National Master’s Examination in General Western Medicine in China (6, 7). In the modern era, medical education faces new tasks and challenges as medical technology develops. To promote medical education and train many high-quality medical talents, medical teaching reform is a top priority.

Over the decades, physiology research has developed a certain scale and character. There is no shortage of macroscopic descriptions of physiology research, most of which are based on specific perspectives. Several studies have explored specific areas of physiology education. To assess teaching methods, Li et al. highlighted the importance of clinical knowledge and experience on reform of teaching preclinical pathophysiology (8). Emery et al. sought to examine the relative importance of environmental influences and individual characteristics on learner-centered teaching practices across institutions (9). Semsar et al. described the development of a new, freely available, online, programmatic-level assessment tool termed Measuring Achievement and Progress in Science in Physiology (Phys-MAPS) to evaluate student learning of core physiology concepts at multiple time points during an undergraduate physiology program, thus providing a valuable longitudinal tool to gain insight into student thinking and aid data-driven reform of physiology curricula (10). Sherer et al. focused on integrating histology and physiology into one course, increasing the two subjects’ connection to clinical medicine, and assessing support for integration and state-of-the-art teaching by comparing students’ attitudes and academic performance in the reformed curriculum (11). To assess teaching content, Ma et al. visualized research hotspots and trends in pyroptosis using bibliometric analysis to understand future developments in basic and clinical research (12). Wang et al. used the bibliometric method to focus on the mechanisms of oxidative stress, neuroinflammation, and autophagy in Parkinson’s disease using animal models (13). Wang et al. summarized the research status of butyrophilins (BTNs), a unique family of immunoglobulins, and their relationship with lung and breast cancers using bibliometrics and bioinformatics methods (14).

The evolution of sub-disciplines and specific research directions in physiology is becoming clearer due to the rapid development of information technology and bibliometrics. However, hot spots and research frontiers in this field are still unclear. Specifically, there is a lack of research on physiology teaching reform (The abbreviation is unified as PTR in this study.). Scientometrics is a branch of informatics that quantitatively analyzes patterns in scientific literature in order to understand emerging trends and the knowledge structure of a research field. Science mapping tools typically use scientific publications as input and generate interactive visual representations of complex structures for statistical analysis and visual exploration (15, 16). CiteSpace is a Java-based application to analyze and visualize hot spots and research frontiers in the scientific literature of a discipline or knowledge domain over a certain period using metrology, co-occurrence analysis, and cluster analysis (15, 17, 18). VOSviewer is software for creating, visualizing, and exploring maps based on web data (19). In addition, it can be used to identify productive journals, co-cited journals, authors, co-cited authors, and related knowledge graphs based on bibliographic data. In the present study, we aimed to analyze hot spots and research frontiers in physiology teaching reform (PTR) using CiteSpace and VOSviewer. We hope the findings will help us understand the future development of basic teaching and clinical research.

## Materials and methods

### Data collection

Data were retrieved from the Web of Science Core Collection (WoSCC) database on May 22, 2022. The WoSCC database covers resources dealing with various branches of science and is regarded as the most influential database^1^. “Physiology” and “Teaching and learning reform” were set as the search terms for articles published between January 1, 2012, and December 31, 2021. The language was restricted to English and the article type was limited to article or review. Table 1 shows the detailed search strategy, including the inclusion and exclusion criteria.

**Table 1:** Search strategy of physiology teaching reforms.

Set	Search query	Publications
#1	(((((((((TS = (Physiology)) OR TS = (Electrophysiology)) OR TS = (Cardiac Electrophysiology)) OR TS = (Endocrinology)) OR TS = (Neuroendocrinology)) OR TS = (Neurophysiology)) OR TS = (Physiology, Comparative)) OR TS = (Psychophysiology)) OR TS = (Neuropsychology)) OR TS = (Psychoneuroimmunology)	238,421
#2	((TS = (teaching)) OR TS = (education)) OR TS = (teach)	696,099
#3	(#1) AND (#2)	5,836
#4	(#3) and Editorial Materials or Meeting Abstracts or Early Access or Proceedings Papers or Book Chapters or Letters or Biographical-Items or Corrections or Reprints (Exclude – Document Types) and Spanish or German or French or Portuguese or Turkish or Czech or Polish or Dutch or Hungarian or Russian or Italian or Japanese or Korean (Exclude – Languages)	2,908
#5	(#4) and 2021 or 2020 or 2019 or 2018 or 2017 or 2016 or 2015 or 2014 or 2013 or 2012 (Publication Years)	2,882

### Bibliometrics and visualization analysis

The “full record and cited references” for the articles identified through the search were exported in “plain text” or “tab delimited” format, named “download_XXX.txt,” and then imported into VOSViewer 1.6.18 and/or CiteSpace 6.1.R2 for further analysis.

We used CiteSpace 6.1.R2 to analyze and visualize trends of high-frequency keywords, dual-map of journals, co-cited references, and citation bursts for references (20, 21). Data analysis was followed by time slicing, thresholding, modeling, pruning, merging, and mapping. The selection criteria of CiteSpace uses a modified g-index per slice (1 year per slice from January 2012 to December 2021). To include more or fewer nodes, we set the scale factor as *k* = 25, the filter criteria as pruning (minimum spanning tree, pruning sliced networks, and the merged network), and the selection criteria (top *N* = 50). For all other settings, the defaults were used (22).

Similarly, we used VOSviewer to identify productive journals, co-cited journals, authors, co-cited authors, and the related knowledge-maps based on bibliographic data. After data cleaning, synonym merging, and deletion of meaningless terms, the high relevance score tendencies in terms of analysis of co-author and keywords were calculated by the fractional counting method (23, 24) The thresholds (T) of authors and countries/regions and organization were set as 5 (25), which are marked in the corresponding tables and figures.

Moreover, we used Microsoft Office Excel 2019 and Origin 2018 to manage the database and fit the analysis. We obtained the 2019 impact factor (IF) and JCR division of journals from the Web of Science InCites Journal Citation Reports^2^ on October 25, 2021.

## Results

### Annual growth trends and distribution of articles

**Figure 1:**
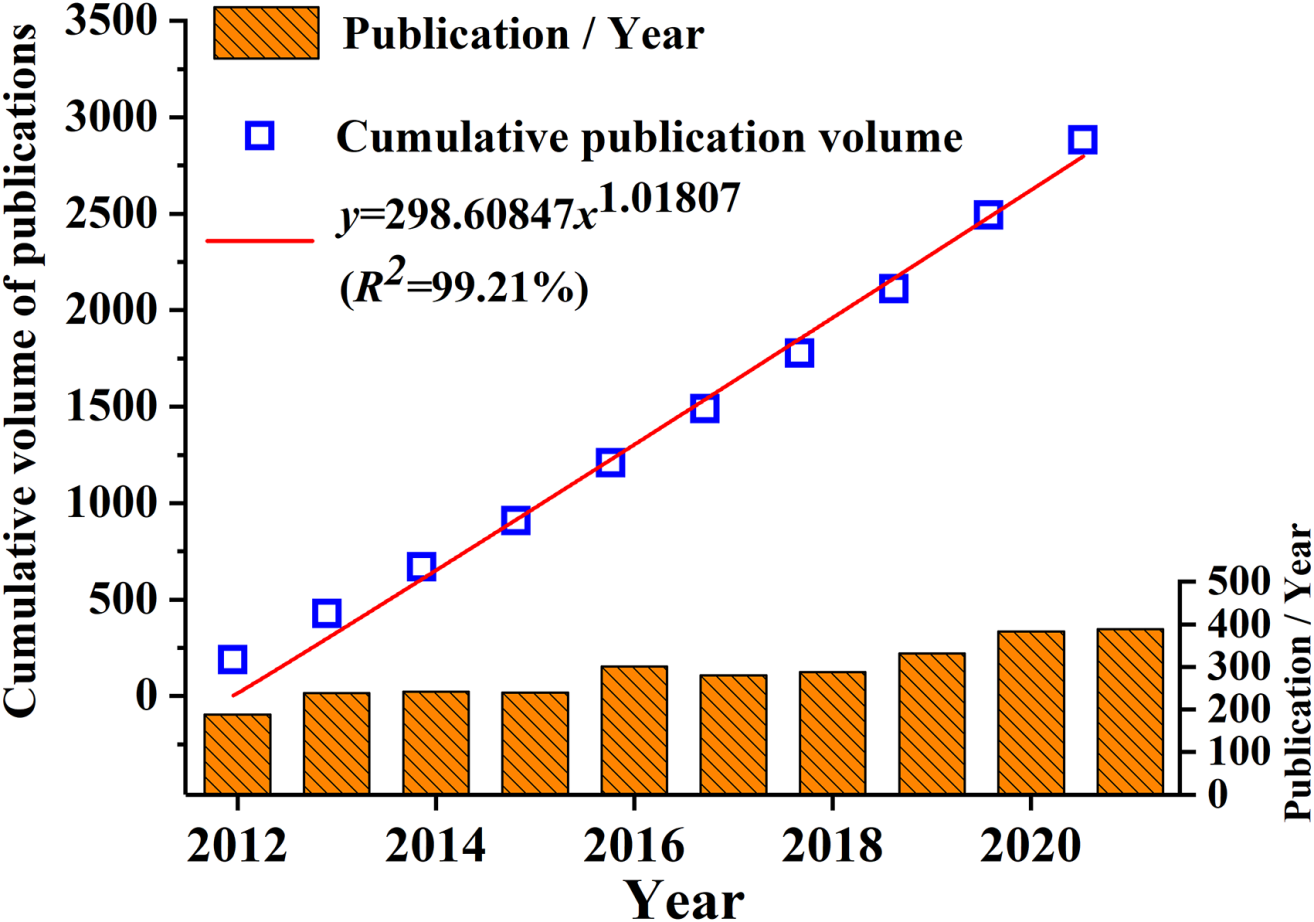
Annual trend of Physiology teaching reform research.

By applying the data collection strategy described above, we obtained 2,882 papers with a total of 128,732 references; this does not include any duplicates. The collected papers were imported into CiteSpace and filtered to identify 2,568 as unique source articles in WOS. The annual growth trend and distribution of the articles was then analyzed. As shown in Figure 1, PTR-related articles showed an annual upward tendency with some fluctuations per year. A descriptive fitting analysis was performed on the cumulative annual volume of publications from 2012 to 2021. The rapidly increasing trend conforms to the formula: *y* = 298.60847x^1.01807^ (*R*^2^ = 99.21%). The yearly output of related publications increased the most significantly (by over 300 publications) from 2019 to 2021, indicating that more and more researchers are beginning to pay attention to this field.

### Co-authorship and co-cited authors

**Figure 2:**
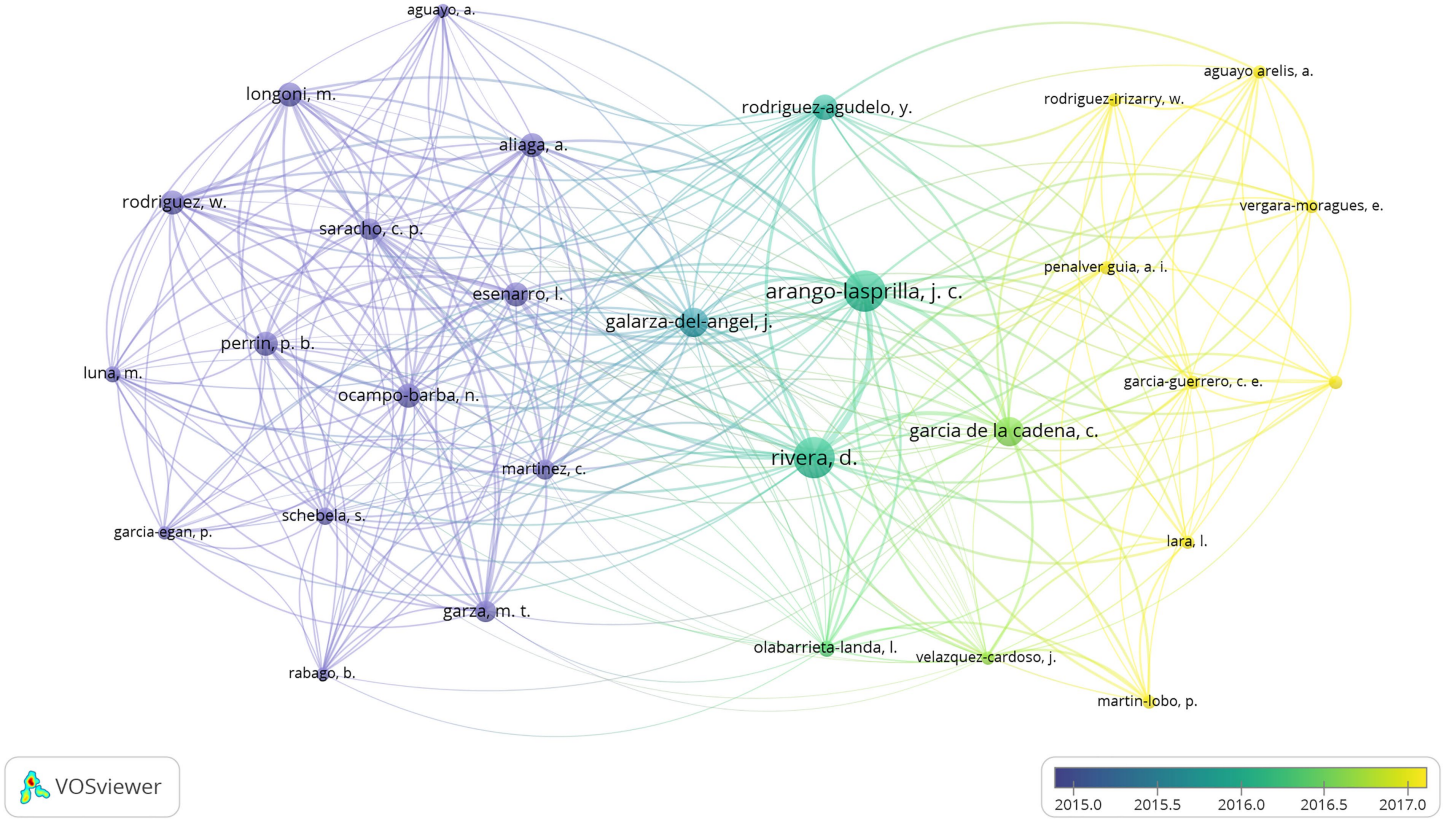
The over-lay map of co-authors in Physiology teaching reforms.

For the co-authorship results, a total of 13,895 authors were involved in PTR. Of these, 70 authors published more than 5 articles (T ≥ 5), which were included to build a network map of authors (Figure 2). The co-authors (*n* = 29) of the 70 authors with 5 published articles were used to make an overlay visualization map; this type of knowledge-map can clearly present high-frequency co-authors by year. The size of the node reflects the author’s co-occurrence frequencies, while the link indicates the co-occurrence relationship between authors. For each of the 70 authors, the total strength of the co-authorship links with other authors was calculated. *Arango-Lasprilla, JC.* and *Rivera, D.* both published the most papers (*n* = 21), followed by *Nijs, J* (*n* = 19), *Louw, A* (*n* = 18), and *Meeus, A* (*n* = 14) (Table 2). Co-cited authors are authors who have been co-cited together in a range of publications. A total of 76,781 co-cited authors were involved in this research area. Of these, 215 co-cited authors published over 20 articles (T ≥ 20). *Moseley, GL* (*n* = 336) ranked first, followed by *Louw, A* (*n* = 285), *Wechsler, D* (*n* = 285), *Michael, J* (*n* = 169), and *Stern, Y* (*n* = 143). The remaining five top authors had 138 to 115 co-citations (Table 2).

**Table 2:** The top 10 co-authors and co-cited authors of physiology teaching reform research.

Rank	First-author	Documents	Citations	First-author	Co-citations
1	Arango-Lasprilla, J. C.	21	321	Moseley, GL	336
2	Rivera, D.	21	321	Louw, A	285
3	Nijs, JO	19	492	Wechsler, D	285
4	Louw, Adriaan	18	457	Michael, J	169
5	Meeus, Mira	14	415	Stern, Y	143
6	Galarza-del-Angel, J.	13	255	World health organization	138
7	Garcia de la Cadena, C.	13	165	Lezak, MD	134
8	Puentedura, Emilio J.	12	418	Nijs, J	130
9	Rodriguez-Agudelo, Y.	11	198	Folstein, MF	123
10	Aliaga, A.	10	246	Ardila, A	115

### Co-institution and co-country

**Figure 3:**
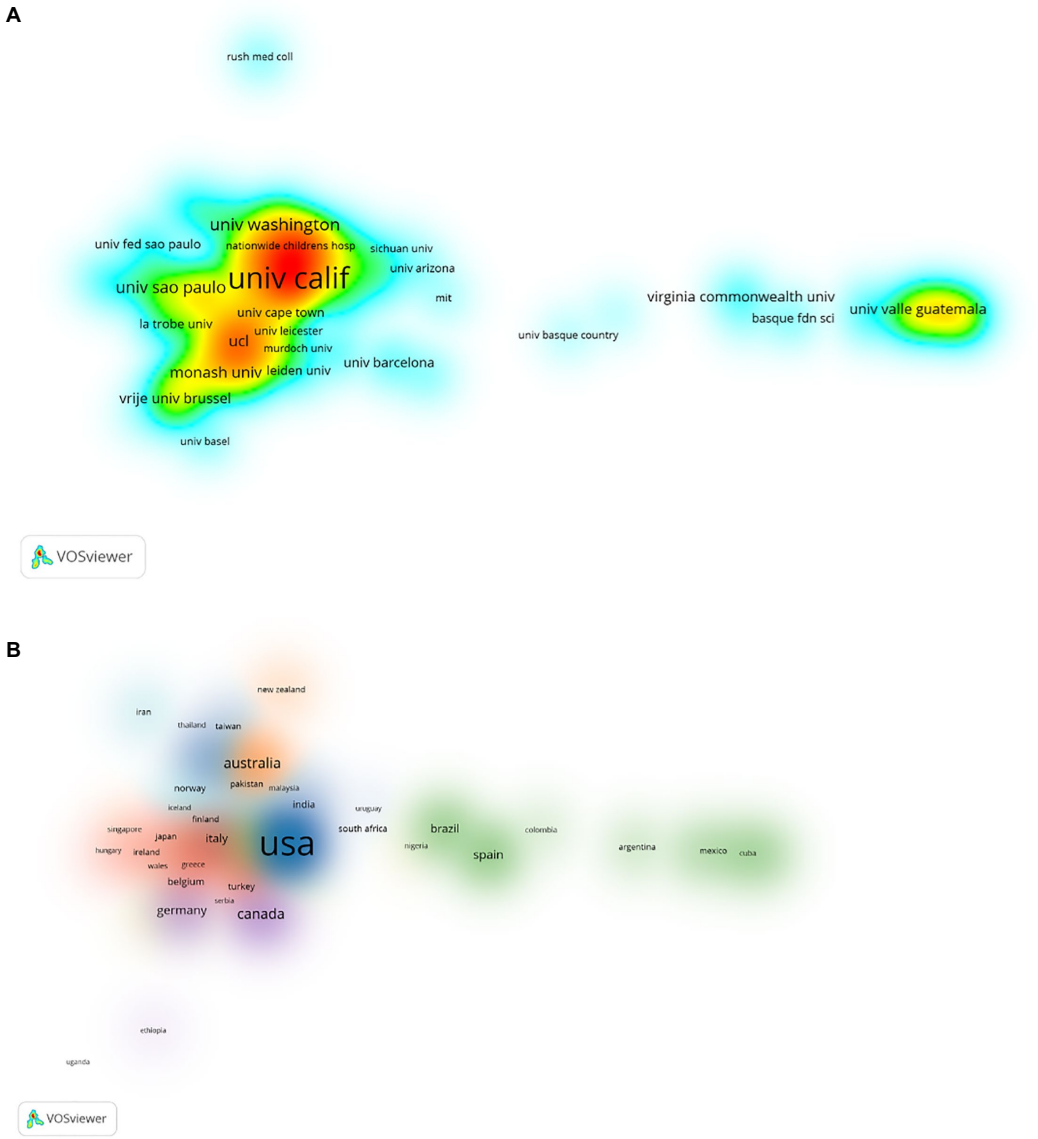
The density map of co-organizations **(A)**. The density map of co-country in Physiology teaching reforms **(B)**.

A total of 4,072 organizations were involved in PTR publishing. Of these, 328 organizations published more than five articles (T ≥ 5). These were included to build a network density map of countries (Figure 3A). The size of the word, the size of circles, and the opacity of yellow are positively related to the co-occurrence frequency. For each of the 328 countries, the total strength of the co-authorship links as calculated. University of California published the most papers (*n* = 118), followed by Harvard University (*n* = 84), Mayo Clinic, University of Toronto (*n* = 42), Johns Hopkins University, University of Colorado, and University of Pennsylvania (*n* = 36, Table 3). A total of 120 countries were involved in PTR research, of which 67 countries published more than five articles (T ≥ 5). These were used to build a network map of country, and different colors are used to distinguish national clusters (Figure 3B). For each of the 67 countries, the total strength of the co-authorship links was calculated. The United States published the most papers (*n* = 1,266) followed by England (*n* = 237), Australia (*n* = 226), Canada (*n* = 212), Spain (*n* = 150), and China (*n* = 139).

**Table 3:** The top 10 co-institution of physiology teaching reform research.

Rank	Organization	Documents	Citations
1	University of California	118	1758
2	Harvard University	84	1,609
3	Mayo clinic	42	661
4	University of Toronto	42	1,329
5	Washington University	37	775
6	The Johns Hopkins University	36	821
7	University of Colorado	36	796
8	University of Pennsylvania	36	1,436
9	Northwestern University	35	709
10	Stanford University	34	742

## Keyword co-occurrence, clusters, and evolution

**Figure 4:**
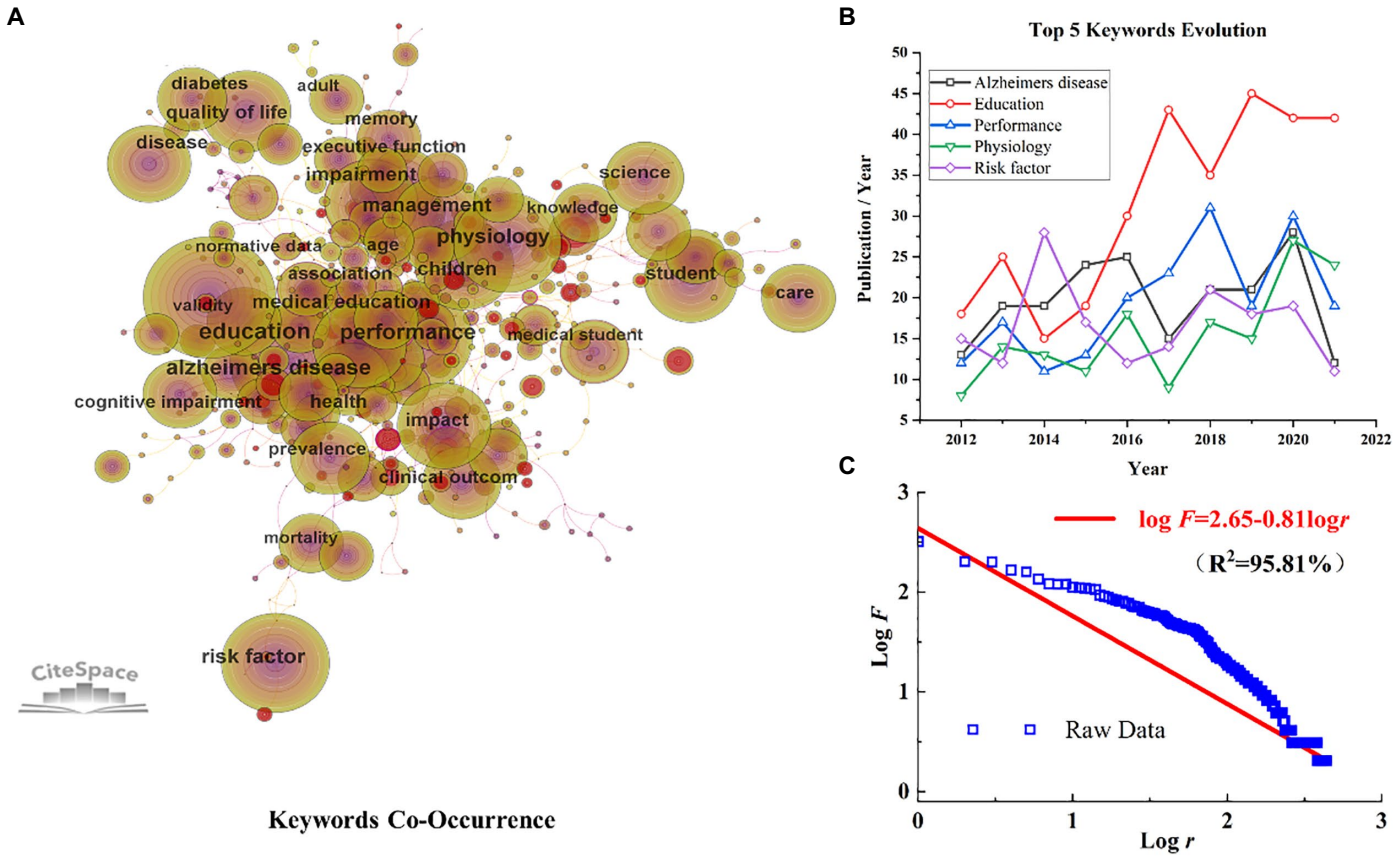
**(A)** The network of keywords co-occurrence. **(B)** The evolution of Top 5 keywords. **(C)** *Zipf*’s law data fitting of key words in physiology teaching reform.

Keywords provide a high-level summary of articles. High frequency and high centrality keywords often reflect hot research topics in a field. The 2,568 unique records identified in WOS were analyzed using CiteSpace. We analyzed publications with a time slicing of 1 year and the top 20 levels of most cited or occurring items from each slice (Figure 4). The trajectory of relevant research is shown as a hybrid network of keyword co-occurrence from the titles and abstracts. Nodes in different maps represent keywords. The size of the nodes indicates the frequency of occurrence or citation, while the color of nodes indicates the occurrence or citation years. Red circles indicate articles with keyword bursts, i.e., rapid increases in publication count. By analyzing the frequency and centrality of keywords, research frontiers can be identified. The network map of keywords consists of 439 nodes and 1,693 links. “Education” has a high frequency and centrality, which means that reform of physiology teaching focuses on students’ practical performance in terms of educational performance and effectiveness (Figure 4A). Similarity, the terms “Alzheimer’s disease” and “performance” are high frequency keywords, reflecting that physiological research about Alzheimer’s disease is a hot topic in current research.

The annual evolution of the top five hot topics with a trend of floating growth is displayed in Figure 4B. The data were quantified and fitted to derive a clearer picture of the evolutionary pattern of the topical vocabulary in physiological research (Figure 4C). The increasing trend in physiological research obeys *Zipf*’s Law ( logf=2.65−0.81logr , *R*^2^ = 95.81%), which is an empirical law of great ubiquity. The top 20 keywords with a high frequency and centrality are presented in Table 4. Interestingly, the term “damage” has a higher centrality which is presumably closely related to the pathological manifestation of brain damage in Alzheimer’s disease.

**Table 4:** Top 20 keywords of physiology teaching reform research in terms of frequency and centrality.

Rank	Keyword	Freq (Count)	Centrality	Rank	Keyword	Freq (Count)	Centrality
1	Education	312 (10.83%)	0.00	11	Student	108 (3.75%)	0.01
2	Alzheimer’s disease	197 (6.84%)	0.03	12	Quality of life	107 (3.71%)	0.02
3	Performance	195 (6.77%)	0.01	13	Impact	105 (3.64%)	0.01
4	Risk factor	162 (5.62%)	0.01	14	Health	103 (3.57%)	0.03
5	Physiology	156 (5.41%)	0.02	15	Science	91 (3.16%)	0.02
6	Management	132 (4.58%)	0.00	16	Clinical outcom	88 (3.05%)	0.00
7	Medical education	118 (4.09%)	0.02	17	Association	87 (3.02%)	0.07
8	Children	117 (4.06%)	0.06	18	Diabetes	83 (2.88%)	0.01
9	Impairment	117 (4.06%)	0.12	19	Age	81 (2.81%)	0.05
10	Disease	109 (3.78%)	0.00	20	Executive function	79 (2.74%)	0.02

**Figure 5:**
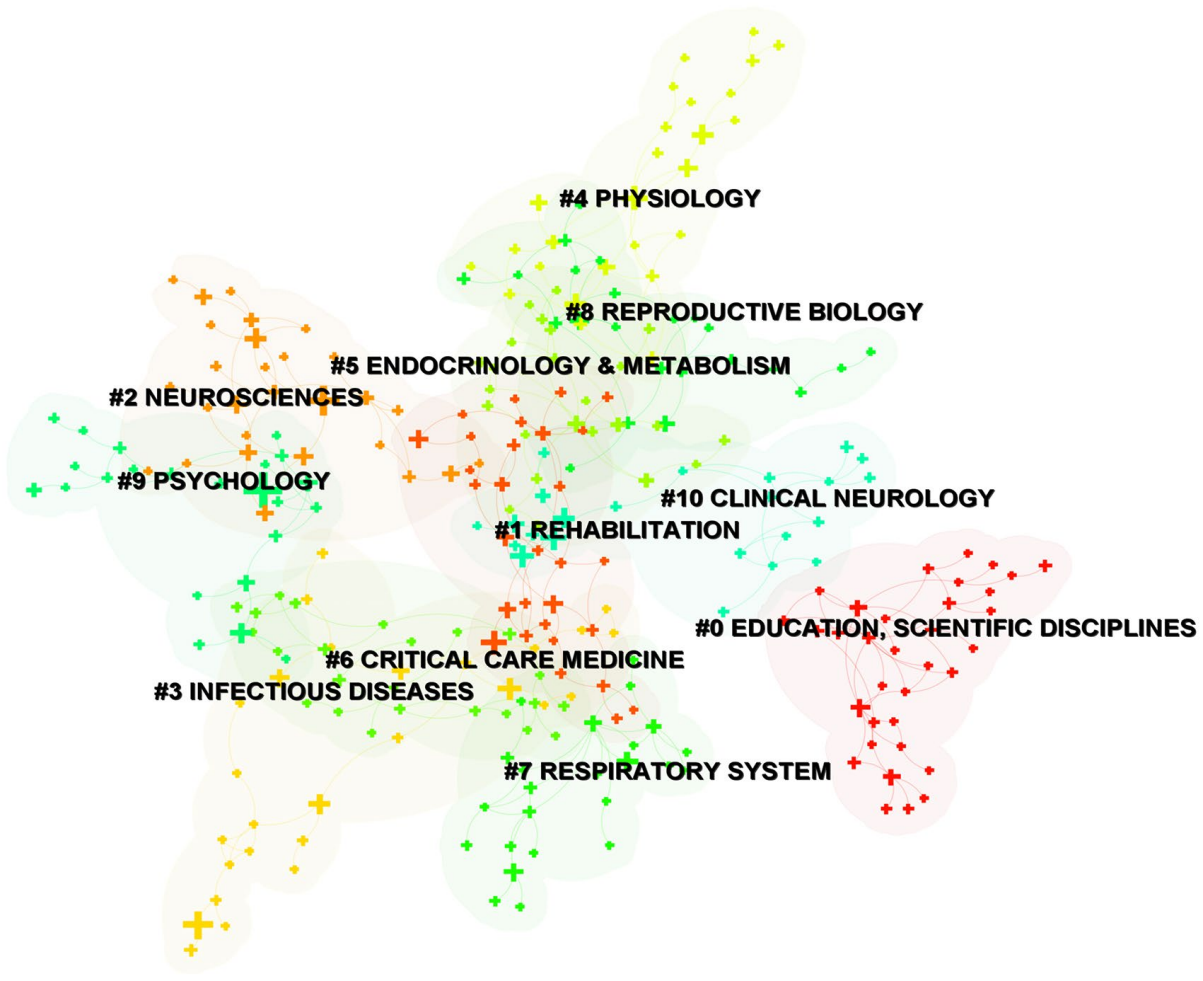
The network of keywords clusters in subject categories.

Cluster analysis can reveal the knowledge structure of a research field (26). To identify the disciplines to which the hot topics belong, we performed a subject clustering analysis of the keywords after simplification by Pathfinder network scaling. According to the link strength of term co-occurrence, the network was divided into 11 clusters (Figure 5). It is highly homogeneous and the most salient connections are between the terms in one cluster. Each article indexed by the Web of Science is assigned one or more subject categories. The most common category is Education (Scientific disciplines), which is also the largest cluster with 34 co-occurrence terms: assessment tool, academic stress, academic achievement, Blooms Taxonomy, Angiography, etc. Cluster 0 is followed by Rehabilitation (Cluster 1), Neurosciences (Cluster 2), and Infectious Disease (Cluster 3). Cluster 1 (Red-orange) includes 30 terms: Boston Naming Test, Motor, Physiotherapy Student, Head Injury, Physiotherapy, etc. Cluster 2 (orange) contains 28 terms: Alzheimer’s Disease, Mild Cognitive Impairment, Life, etc. Cluster 3 (yellow) has 27 terms: Epidemiology, Prevention, Risk Factor, etc. Although Physiology, Endocrinology & Metabolism, Critical Care Medicine, Respiratory System, Reproductive Biology, Psychology, and Clinical Neurology are much smaller clusters, they are marked for reference. The sub-disciplines around physiology remain an important part of the exploration of PTR.

**Figure 6:**
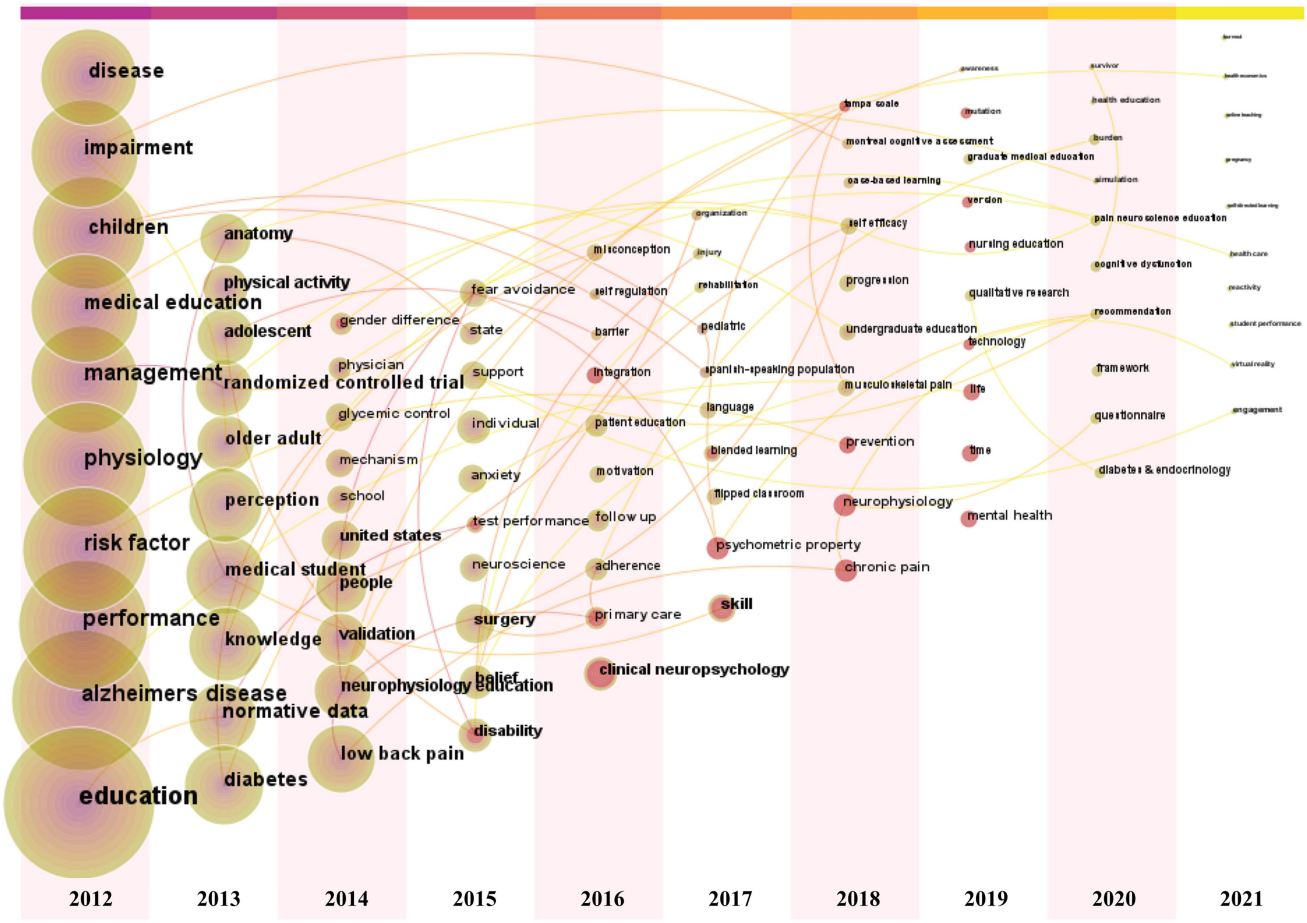
The annual top 10 keywords of physiology teaching reforms were showed by keywords time zone view in 2012–2021.

The keyword time zone view was designed by CiteSpace to clearly show the evolution of high frequency keywords. Keywords are located in the year they first co-occurred and the color of the links represents the first year two keywords appear simultaneously. Consequently, we added the top 10 high frequency keywords each year from 2012 to 2021 to supplement the time zone map (Figure 6). The size of the cross and word reflects the co-occurrence frequencies, while the link indicates the co-occurrence relationship. The colors of the node and line represent different years; colors vary from purple to red as the years go from 2012 to 2021. Of these, Clinical Neuropsychology may have experienced a turning point with a high count in 2016.

**Figure 7:**
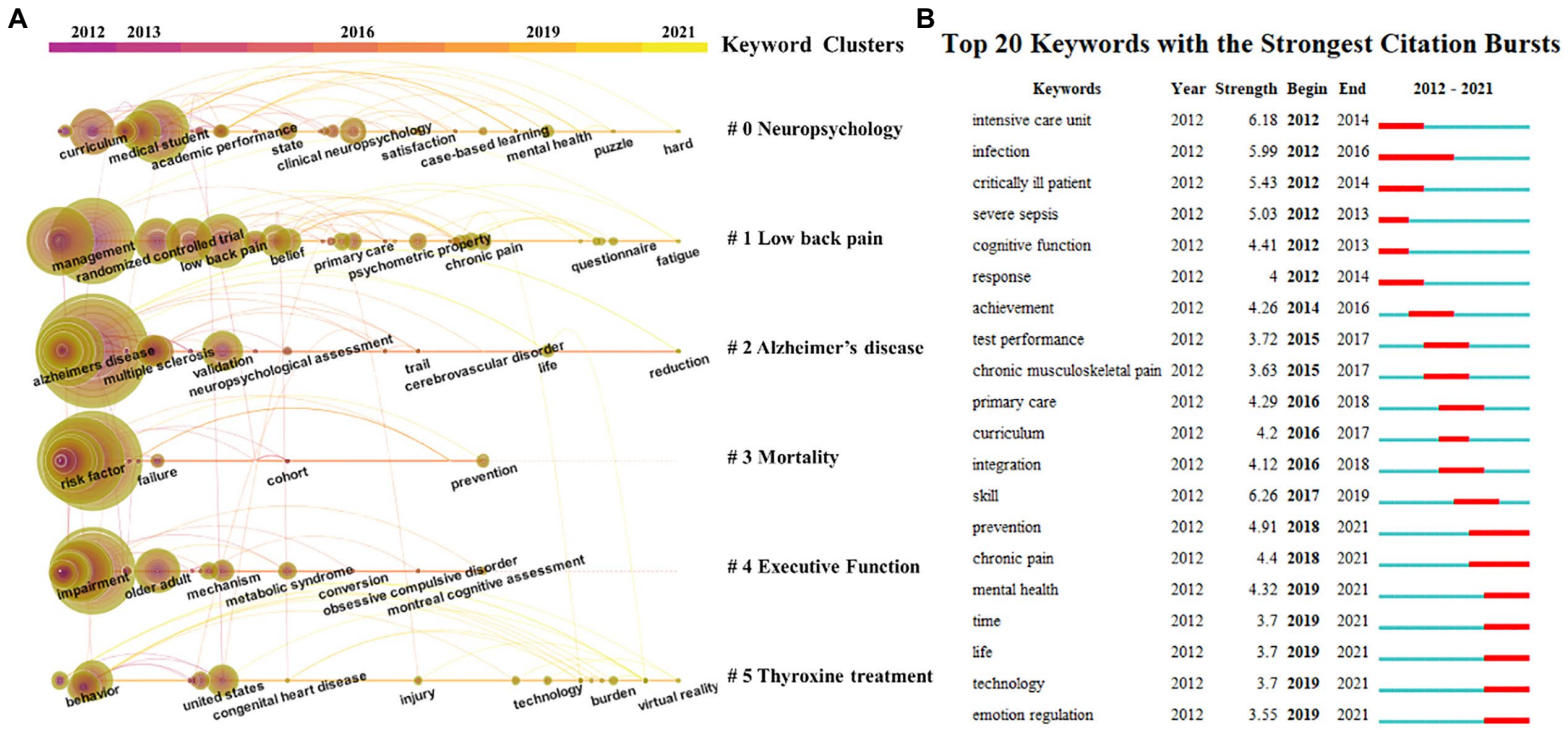
**(A)** The keywords of physiology teaching reforms were showed in keywords timeline view in 2012–2021. **(B)** The top 20 keywords with citation bursts.

The keyword clusters were also displayed in the keyword timeline view (Figure 7A). The six keyword clusters included Neuropsychology, Low Back Pain, Alzheimer’s Disease, Mortality, Executive Function, and Thyroxine Treatment. The keywords with citation bursts appeared in 2012, 2013, 2016, 2019, and 2021. The top 20 keywords with citation bursts are presented in Figure 7B. The blue line indicates the time interval, while the red line indicates the time period when the keyword had a burst (17). The keywords Intensive Care Unit had the strongest citation burst and appeared in 2012, indicating the importance of medical facilities in physiological research. The most recent keywords with citation bursts that occurred in 2019 were Mental Health and Emotion Regulation, demonstrating the focus on mental and neurological health in recent years.

### Journals and co-cited journals

We used VOSviewer to conduct co-citation and co-cited journal analyses, finding the most active and influential journals in the field. The results described that the 1,267 references were published in 466 academic journals. *Advances in Physiology Education* published the most papers (309, 10.72%), followed by *Clinical Neuropsychologist*, *BMJ Open*, *Archives of Clinical Neuropsychology*, *Anatomical Sciences Education*, *BMC Medical Education*, *Critical Care Medicine*, *Applied Neuropsychology-Adult*, *Journal of Alzheimer’s Disease*, and *Physiotherapy Theory and Practice*. Among the top 10 journals, “*Critical Care Medicine*” was in the Q1 JCR division, five were in the Q2 JCR division, and two had an impact factor (IF) greater than five (Table 5).

**Table 5:** The top 10 journals of physiology teaching reform research.

Rank	Journal	*N* (%)	IF (2020)	JCR division	Country
1	Advances in physiology education	309 (10.72%)	2.288	Q3/Q4	United States
2	Clinical neuropsychologist	62 (2.15%)	3.535	Q2	Netherlands
3	Bmj open	44 (1.53%)	2.692	Q2	England
4	Archives of clinical neuropsychology	43 (1.49%)	2.813	Q3/Q4	England
5	Anatomical sciences education	37 (1.28%)	5.598	Q2	United States
6	Bmc medical education	37 (1.28%)	2.463	Q2	England
7	Critical care medicine	33 (1.15%)	7.598	Q1	United States
8	Applied neuropsychology-adult	31 (1.08%)	2.248	Q3/Q4	United States
9	Journal of Alzheimer’s disease	29 (1.01%)	4.472	Q2	Netherlands
10	Physiotherapy theory and practice	26 (0.9%)	2.279	Q3	England

**Figure 8:**
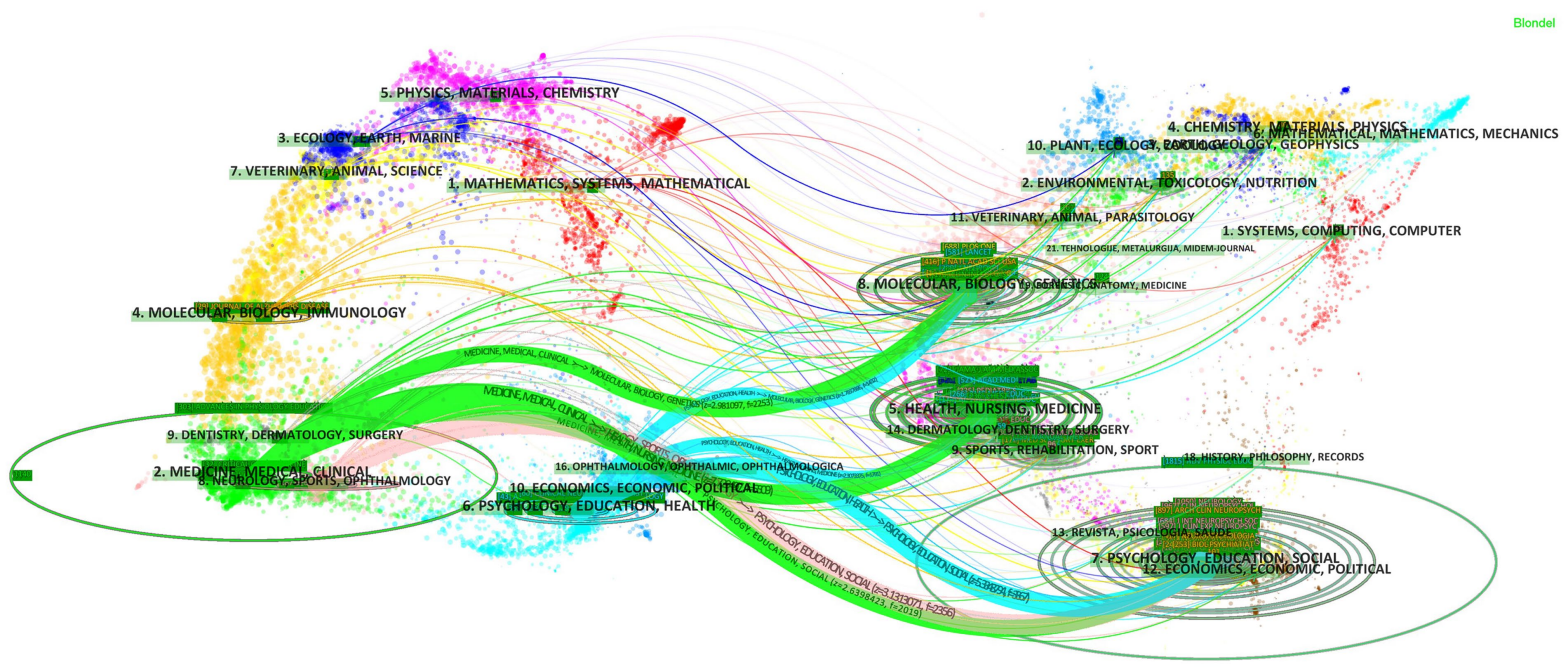
The dual-map overlay of journals related to physiology teaching reform research. The citing journals were at left, the cited journals were on the right, and the colored path represents citation relationship.

Among the 4,781 co-cited journals, two journals had more than 1,000 citations. As shown in Supplementary Table S1, *Advances in Physiology Education* had the most co-citations (2,177, 75.54%) followed by *Critical Care Medicine* and *Clinical Neurophysiology*. Among the top 10 co-cited journals, four were in the Q1 JCR division with an IF greater than seven and five were from the United States. The dual-map overlay of journals reflects the topic distribution of academic journals (18) (Figure 8). The three primary citation paths colored green were identified, meaning that studies published in Psychology/Education/Social journals, Health/Nursing/Medicine journals, and Molecular/Biology/Genetics journals were mainly cited by studies published in Medicine/Medical/Clinical journals. Three primary citation paths colored blue were identified, meaning that studies published in Molecular/Biology/Genetics journals, Health/Nursing/Medicine journals, and Psychology/ Education/Social journals were mainly cited by studies published in Psychology/Education/Health journals. One primary citation path colored pink was identified, which means that the studies published in Psychology/Education/Social journals were mainly cited by studies published in Neurology/Sports/Ophthalmology journals.

### Reference co-citations

**Figure 9:**
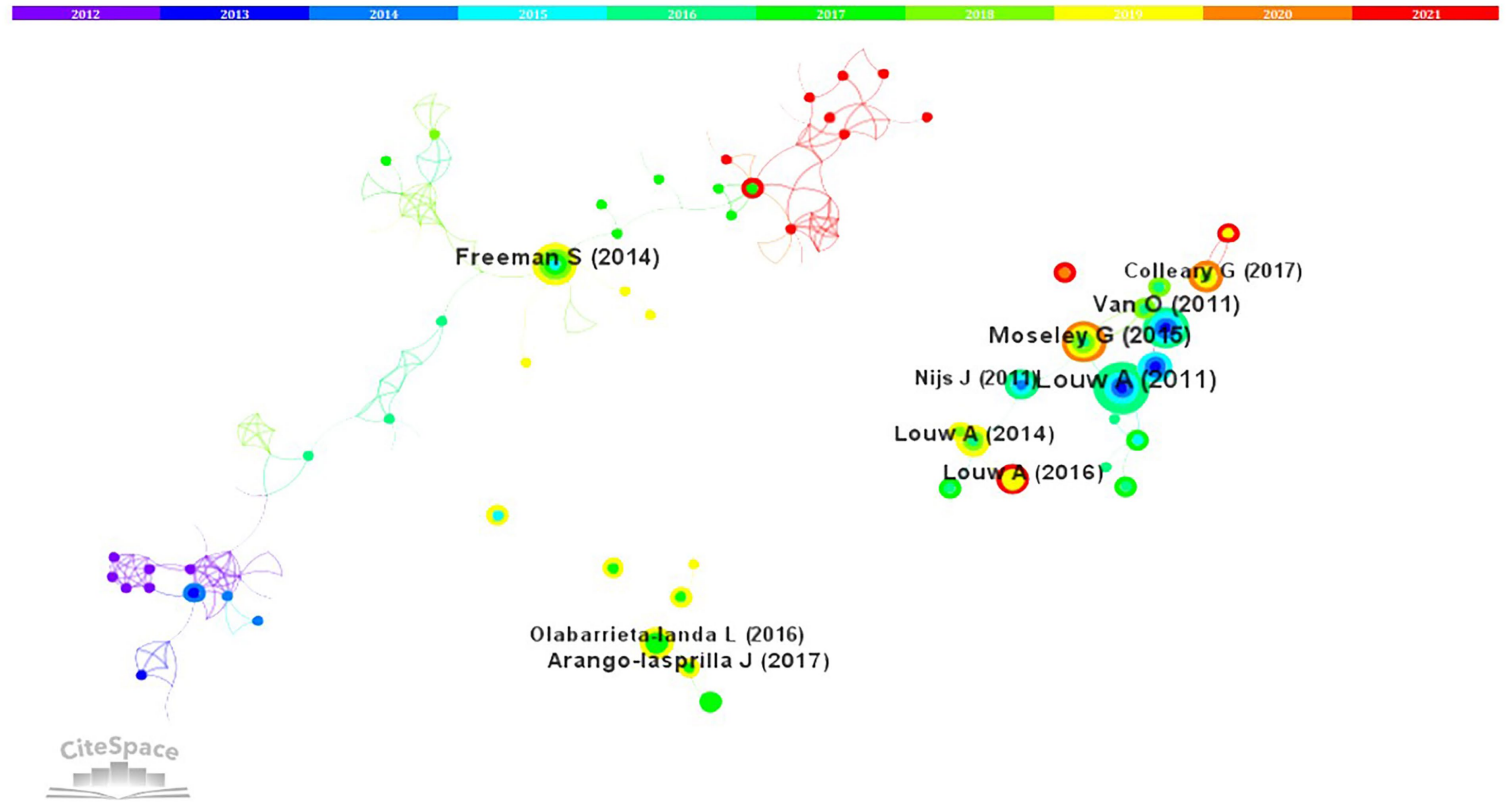
The network of co-cited references in physiology teaching reform research.

We used CiteSpace to examine co-cited references. Figure 9 and Supplementary Table S2 show the top 10 co-cited references with a high frequency and betweenness centrality. The network of co-cited references in PTR research comprised 65,889 references with 495 nodes and 833 links. The top co-cited reference was an article published in *Arch Phys Med Rehab* by Louw et al. entitled “The effect of neuroscience education on pain, disability, anxiety, and stress in chronic musculoskeletal pain.” The article reports the effectiveness of neuroscience education for pain, disability, anxiety, and stress in chronic musculoskeletal pain (27). Many studies examined pain. The reference published by *Moseley* et al. introduced the concept of explaining pain (EP) to help patients understand current thought in pain biology (28). Freeman et al. demonstrated that lecturing maximizes learning and course performance through a meta-analysis of 225 studies of student performance in undergraduate science, technology, engineering, and mathematics (STEM) courses under traditional lecturing versus active learning (29). Van et al. examined whether education about the neurophysiology of pain is accompanied by changes in symptoms, daily functioning, pain beliefs, and behavior in a single-case study (30). Louw et al. noted that the addition of neuroscience education to usual preoperative education results in superior outcomes with regard to pain, function, surgical experience, and health care utilization post-surgery (31, 32). Arango-Lasprilla et al. analyzed the characteristics of individuals working in neuropsychology in Latin America to forge links between neuropsychology and personal background and performance (33). Other highly cited publications were as follows: *Nijs* et al. revealed that pain physiology education can be accomplished by patient education about central sensitization and its role in chronic pain (34). Colleary et al. considered the effect of pain neurophysiology education (PNE) on student physiotherapists (35). Olabarrieta-Landa et al. pointed out that neuropsychology in Spain needs to regulate professional neuropsychology, improve graduate curricula, enhance existing clinical training, develop professional certification programs, validate and create normative data for existing neuropsychological tests, and create new, culturally relevant instruments (36).

**Figure 10:**
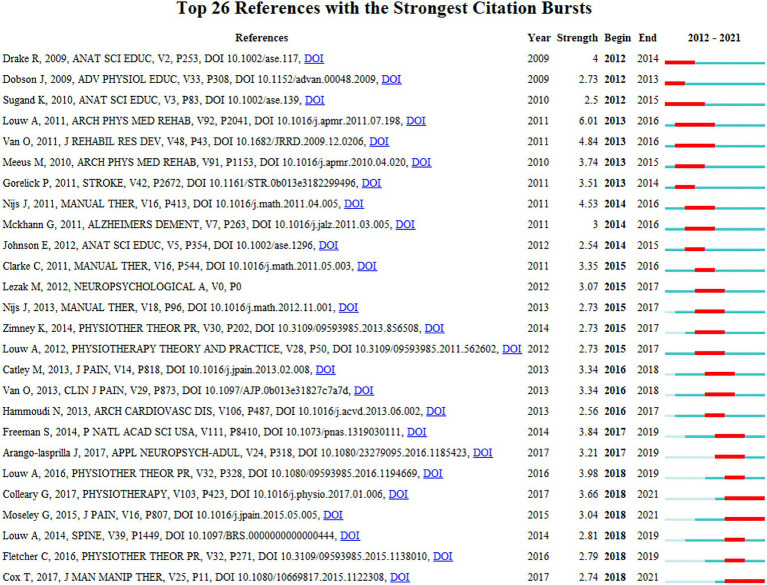
Top 26 references with the strongest citation bursts (sorted by the beginning year of burst). The blue bars mean the reference had been published; the red bars mean citation burstness.

Figure 10 presents the top 26 references with strongest citation bursts, which indicate emerging trends or increasing interests in the field. Generally, the most co-cited references have the strongest citation bursts. The strongest citation bursts ranking showed that 23.08% (6/26) of references had citation bursts in 2018, followed by 2015 (5/26, 19.23%) and 2013 (4/26, 15.38%). Notably, three references (11.54%) had bursts until 2020. The paper with the strongest burstness (strength = 6.01) was entitled “The effect of neuroscience education on pain, disability, anxiety, and stress in chronic musculoskeletal pain” (27), which also was the most co-cited reference published in *Arch Phys Med Rehabil* by Louw et al. in 2011 with a citation burst from 2013 to 2016.

## Discussion

Based on the data from WoSCC database, this study performed a bibliometric analysis of 2,882 PTR-related publications published in 466 academic journals by 13,895 authors in 4072 organizations from 67 countries/regions. Change in annual output is an essential indicator of development in a field. PTR-related articles displayed an annual overall upward tendency with some fluctuations each year from 2012 to 2021. The change does not reflect a clear phasing, but rather a continuous and steady trend of growth. Thus, it is expected that exploration of physiology teaching research will continue to be of interest for long into the future.

Contribution analysis of authors with many co-occurrences or co-cited papers in a specific field is helpful for sorting out key information such as research background and author collaborations. Focusing on co-authors and co-cited authors, we found that 70 authors published more than five articles from 13,895 authors in PTR. Arango-Lasprilla. and Rivera. both published the most papers, while Moseley had the most co-citations at more than 300. These findings imply that an author’s article has a high impact or is a landmark paper in that field during that time period. These results deserve further exploration. Furthermore, the map of authors and co-cited authors provides information about potential collaborators and influential research groups. In the PTR field, researchers actively cooperate within and between institutions. Besides the influence of the authors themselves, the research institutions are an important influencing factor in contributions to a research field. For example, 328 organizations published more than five articles from 4,072 organizations. The University of California published the most papers, which reflects the high importance that the United States attaches to medical education. From a national perspective, the United States, England, Australia, Canada, Spain, and China were the top six productive countries. It is worth noting that the United States was the first and dominant country taking up PTR research in the time frame studied. Largely due to language limitations in database inclusion and screening criteria, the volume of publications from China is slightly lower.

The journals and co-cited journals analysis presented that *Advances in Physiology Education* published the most papers. *Critical Care Medicine* was in the Q1 JCR division. *Anatomical Sciences Education and Critical Care Medicine* each have an impact factor greater than 5. These findings highlight the strong influence and guiding role that these journals play in PTR field. *Advances in Physiology Education* had the most co-citations, *Critical Care Medicine* was second and has an IF of 7.598 (2020) in the JCR Q1 division, and *Clinical Neurophysiology* is third for co-citations. The *Cochrane Database of Systematic Reviews* has the highest IF among the top 10 co-cited journals at 9.266, and thus plays a leading role in the field. Both of these journals are in the Psychology, Education, and Medicine category, which is consistent with the dual-map analysis. The dual-map overlay of journals indicates their topic distribution. The three primary citation paths from Psychology/Education/Social journals, Health/Nursing/Medicine journals, and Molecular/Biology/Genetics journals were mainly cited by studies published in Medicine/Medical/Clinical journals. The three primary citation paths from Molecular/Biology/Genetics journals, Health/Nursing/Medicine journals, and Psychology/ Education/Social journals were mainly cited by studies published in Psychology/Education/Health journals. The one primary citation path from Psychology/Education/Social journals was mainly cited by studies published in Neurology/Sports/Ophthalmology journals. These findings imply that physiological research is focused on basic research, is closely integrated with clinical research, and is gradually integrating the importance of healthy lifestyle; however, research on translational medicine is still limited. Journals in the Q1 JCR division with a high IF accounted for most of the top 10 journals (10%) and co-cited journals (40%). These findings indicate that these journals are interested in and play essential roles in physiological-related research, and thus have greater development space. Notably, Louw et al. published the first co-cited reference, which was on the effectiveness of neuroscience education for pain, disability, anxiety, and stress in chronic musculoskeletal pain. This was also the reference with the strongest citation burstness from 2013 to 2016 published in *Arch Phys Med Rehabil*. This shows that of PTR research is inseparable from basic research and on-going development of basic research is still an important link to teaching reform.

Examining co-occurrence and cluster analysis of keywords can help guide the evolution process and clarify research directions. We analyzed the trajectories, frequency, and centrality of the keyword network. “Education” had a high frequency and centrality, while the terms “Alzheimer’s disease” and “performance” were high frequency keywords, reflecting that physiological research about Alzheimer’s disease is currently a hot topic. Furthermore, it shows that the “BRAIN Initiative” announced by the National Institutes of Health (NIH) (37), which is cognitive impairment-oriented program combined with clinical research and teaching reform, is closely linked and thus a good starting point for reform. Physiology relevant research is still a research hotspot and the overall trend is increasing year by year. According to the link strength of term co-occurrence, the network was divided into 11 clusters with high homogeneity and salient connections. Rehabilitation (Cluster 1), Neurosciences (Cluster 2), and Infectious Disease (Cluster 3) remain an important part of PTR. With mental and neurological health becoming the focus of attention in recent years, clinical neuropsychology may be positioned to take a new turn and grow at a rapid pace.

From our analysis, we can see that research on PTR initially focused on the education method itself. Afterward, basic research in the fields of rehabilitation, neuroscience, and infectious diseases gradually deepened, such as Alzheimer’s disease, Parkinson’s disease, and pain. Notably, a healthy lifestyle supported by clinical guidance has gradually become a hot topic. We are in an important development stage of medicine as research moves toward supporting people’s health. However, in depth basic physiological research is still necessary, which is consistent with the findings of our bibliometric analysis.

Compared with other reviews, our study using CiteSpace provided a visualization of research hotspots and frontiers. However, this study suffers from certain limitations inherent to bibliometrics. Firstly, data were retrieved only from the WoSCC database, and studies not included in the WoSCC may have been missed. Furthermore, for this paper, only articles published in English were included. The large number of articles published in Chinese in the China national knowledge infrastructure (CNKI) database is still a significant reference with comparative research value. Secondly, differences in screening strategies may lead to slightly different results. Nevertheless, we believe that the use of multiple bibliometric tools and quantitative analysis support the reliability of this study, which can provide researchers with a richer source of objective information.

## Conclusion

In summary, research in PTR is experiencing a rapid phase of development with active global collaborations with the United States as the center within the current search scope. Current publications are focused on medicine, education, and neurology. Rehabilitation, neuroscience, and infectious diseases are popular research areas within physiology research. In particular, research into the pathogenesis, treatment strategies, and technological updates of neurological disorders is becoming a hot topic. We also analyzed the data using CiteSpace and VosViewer, which allow for richer results from different perspectives. Compared to traditional reviews, this study provides original and objective insights into research on PTR. We believe that the results of this study will be a useful reference in the future.

## Data availability statement

The raw data supporting the conclusions of this article will be made available by the authors, without undue reservation.

## Author contributions

JX and QM designed this study, performed the analysis, and normalized the pictures. SS and YZ collected the data. JX wrote the original draft. All authors contributed to the article and approved the submitted version.

## Funding

This work was supported by the Higher Education Research Project of Zhejiang Higher Education Society (KT2021067), the Teaching and Research Project of Ningbo University (JYXMXZD2021030), the Ningbo Education Science Planning Project (2021YGH009), and the Fundamental Research Funds for the Provincial Universities of Zhejiang (SJLZ2022001).

## Conflict of interest

The authors declare that the research was conducted in the absence of any commercial or financial relationships that could be construed as a potential conflict of interest.

## Publisher’s note

All claims expressed in this article are solely those of the authors and do not necessarily represent those of their affiliated organizations, or those of the publisher, the editors and the reviewers. Any product that may be evaluated in this article, or claim that may be made by its manufacturer, is not guaranteed or endorsed by the publisher.

## Footnotes

^1^ https://mjl.clarivate.com/help-center

^2^ https://jcr.clarivate.com/jcr/home
