# Supplementary material for: Knowledge domain, research hotspots and frontiers in physiology teaching reforms from 2012 to 2021: A bibliometric and knowledge-map analysis
Source: Front Med (Lausanne). 2023 Mar 20;10:1031713. doi: 10.3389/fmed.2023.1031713 (PMC10067749; doi:10.3389/fmed.2023.1031713)
Supplement: Supplementary file 1 [file Table_1.docx]

Table S1 The top 10 Co-cited journals of Physiology Teaching Reform research.

| Rank | Co-cited Journal | N (%) | IF (2020) | JCR division | Country |
| --- | --- | --- | --- | --- | --- |
| 1 | *Advances in physiology education* | 2177 (75.54%) | 2.288 | Q3/Q4 | UNITED STATES |
| 2 | *Critical care medicine* | 1502 (52.12%) | 7.598 | Q1 | UNITED STATES |
| 3 | *Clinical neurophysiology* | 937 (32.51%) | 3.708 | Q2 | IRELAND |
| 4 | *clinical neuropsychologist* | 723 (25.09%) | 3.535 | Q2 | NETHERLANDS |
| 5 | *Cochrane database of systematic reviews* | 723 (25.09%) | 9.266 | Q1 | ENGLAND |
| 6 | *Anatomical sciences education* | 705 (24.46%) | 5.598 | Q2 | UNITED STATES |
| 7 | *Archives of clinical neuropsychology* | 660 (22.9%) | 2.813 | Q3/Q4 | ENGLAND |
| 8 | *Mayo clinic proceedings* | 657 (22.8%) | 7.616 | Q1 | UNITED STATES |
| 9 | *Critical care* | 583 (20.23%) | 9.097 | Q1 | ENGLAND |
| 10 | *Journal of clinical endocrinology & metabolism* | 552 (19.15%) | 5.958 | Q2 | UNITED STATES |
