# Supplementary material for: Knowledge domain, research hotspots and frontiers in physiology teaching reforms from 2012 to 2021: A bibliometric and knowledge-map analysis
Source: Front Med (Lausanne). 2023 Mar 20;10:1031713. doi: 10.3389/fmed.2023.1031713 (PMC10067749; doi:10.3389/fmed.2023.1031713)
Supplement: Supplementary file 2 [file Table_2.docx]

Table S2 The top 10 Co-cited references of Physiology Teaching Reform research.

| Rank | First Author | Country | Frequency | Centrality | Year | Source | IF |
| --- | --- | --- | --- | --- | --- | --- | --- |
| 1 | Louw A | USA | 21 | 0.01 | 2011 | ARCH PHYS MED REHAB | 3.966 |
| 2 | Moseley G | Australia | 19 | 0.02 | 2015 | J PAIN | 5.828 |
| 3 | Freeman S | USA | 18 | 0.05 | 2014 | P NATL ACAD SCI USA | 11.205 |
| 4 | Van O | Belgium | 17 | 0 | 2011 | J REHABIL RES DEV | 1.277 |
| 5 | Louw A | South Africa | 15 | 0.02 | 2014 | SPINE | 3.468 |
| 6 | Arango-lasprilla J | Spain | 14 | 0 | 2017 | APPL NEUROPSYCH-ADUL | 2.248 |
| 7 | Louw A | USA | 13 | 0 | 2016 | PHYSIOTHER THEOR PR | 2.279 |
| 8 | Nijs J | Belgium | 12 | 0.01 | 2011 | MANUAL THER | 2.622 |
| 9 | Colleary G | UK | 12 | 0.01 | 2017 | PHYSIOTHERAPY | 3.358 |
| 10 | Olabarrieta-landa L | Spain | 12 | 0 | 2016 | CLIN NEUROPSYCHOL | 3.535 |
